# Supplementary material for: Trauma and resilience in an urban clinic for unhoused young adults: A mixed methods study
Source: PLOS Ment Health. 2025 Sep 5;2(9):e0000392. doi: 10.1371/journal.pmen.0000392 (PMC12798583; doi:10.1371/journal.pmen.0000392)

**S1 Text:** **Adverse Childhood Experience Questionnaire for Adults, Philadelphia Expanded ACES Questionnaire, PHQ-9, and Brief Resilience Scale (BRS)**

Fig A: ACES Questionnaire for Adults
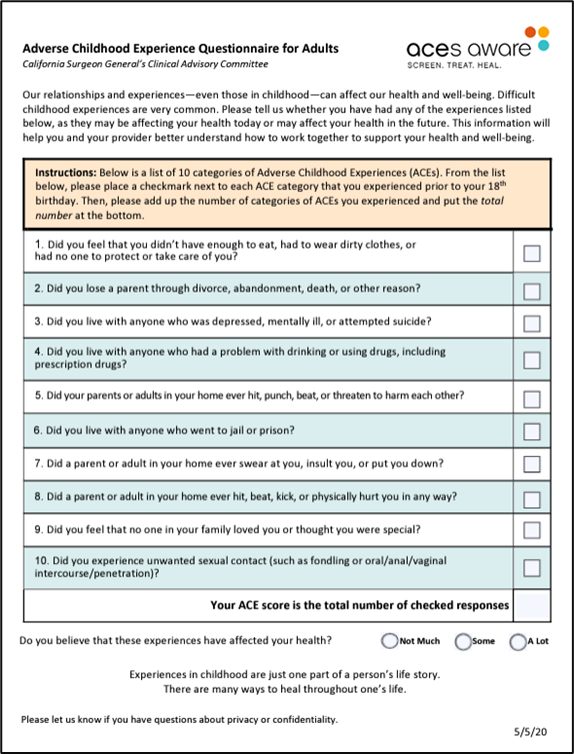


Fig B: Philadelphia Expanded ACE Questionnaire


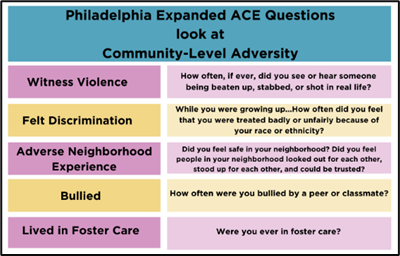


Fig C: PHQ-9


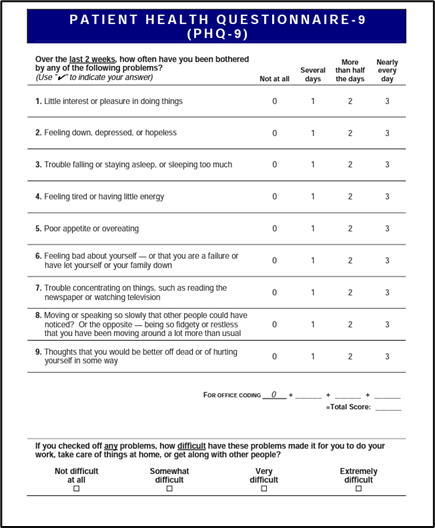


Fig D: Brief Resilience Scale (BRS)


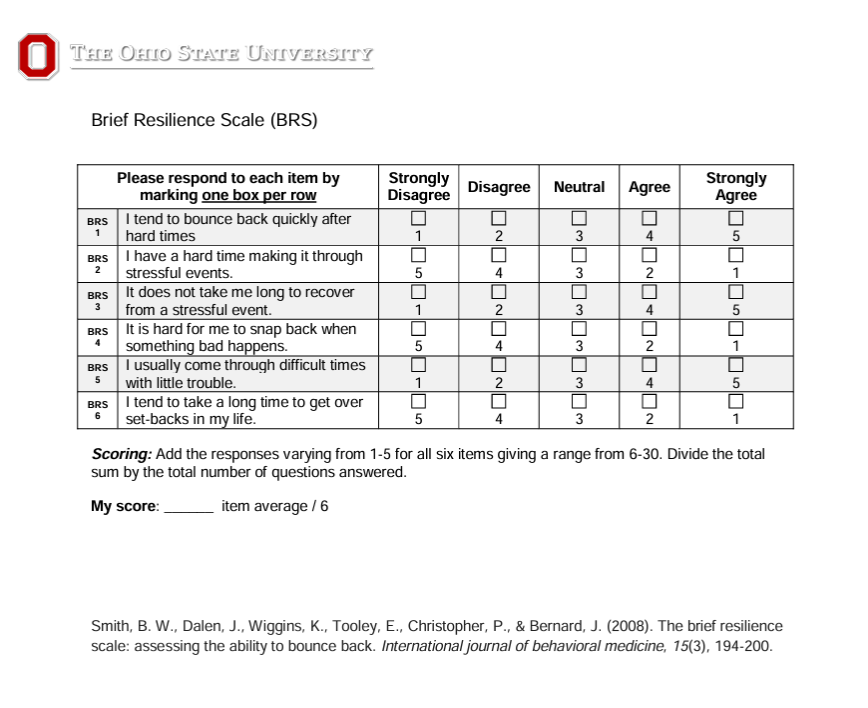

Supplement: S1 Text — (DOCX) [file pmen.0000392.s001.docx]
