# Supplementary material for: Trauma and resilience in an urban clinic for unhoused young adults: A mixed methods study
Source: PLOS Ment Health. 2025 Sep 5;2(9):e0000392. doi: 10.1371/journal.pmen.0000392 (PMC12798583; doi:10.1371/journal.pmen.0000392)
Supplement: S1 Table — (DOCX) [file pmen.0000392.s002.docx]

**S1 Table**

**Core reason for first instance of housing instability.**

| **Core Reason for First Instance of Housing Instability** | **Participant IDs (n=28)** |
| --- | --- |
| Kicked out by family/foster family prior to 18 | 4 – When I was in 7^th^ or 8^th^ grade, my dad kicked me out so ITable slept under bridges. |
|  | 5 – When my foster family found out I identified as trans male, they kicked me out. I moved to my girlfriend’s house to finish high school. |
| Actively decided to leave housing prior to 18 | 2 – I decided to run away last August (2022) because I was tired of being treated like a baby. Now that I’ve gone away from them, I can wear whatever I want. My parents dropped me off at [shelter] |
|  | 20 – I became homeless at 17. Growing up my house had domestic violence and my siblings and mom were violent towards me. I also experienced sexual assault from someone I knew but my family doesn’t know. |
|  | 22 – Housing became unstable when I was 17 so I lived in my car. |
|  | 23 – I tried to overdose on several different types of meds. Mom made me stop taking meds and threatened to kick me out. I stopped going to high school at age 17. I lived at [shelter] between April and May, now I’m currently in an apartment with my mom and stepdad. |
| Actively decided to leave housing after 18 | 3 – At some times I lived with friends, but we had an argument one day and was kicked out. Instead of living with my grandparents I chose to be homeless. |
|  | 21 – My parents were divorced, and I lived with mom growing up. After I turned 18, I lived on the streets for almost 2 years. I didn’t finish high school. |
|  | 24 – I lived with my dad until age 18 when he passed. I have no contact with the rest of my family because of drama. I moved in with my aunt and later went to the shelters. |
| Unable to afford or find housing after 18 | 9 – In May 2022, I lost my job due to the pandemic and became homeless. I moved in briefly with my mother. Then my partner found [shelter] so we moved there with my child [6 years old].  28 – When I Turned 18, I started hormone replacement therapy (HRT) in secret. My mom found out in April and kicked me out. |
|  | 11 – My mom and stepmom showed violence towards me, and Dad abused alcohol. Eventually I finished high school, with plans to attend [university] or [university] in the fall. I moved into my grandmother’s place, tried leaving to [city] but this was a negative experience so I ended up at the shelter. |
|  | 14 – My spouse and I lived with my parents until I turned 19. I separated from my spouse early February, came to [city] and became unhoused. |
|  | 15 – I was most recently at [shelter] where I met my new partner. We now live in my partner’s tent with my service animal. We have current issues with getting housing; our dog has been denied entry and the case worker told us to “sleep in the kennel” with my dog. |
|  | 16 – I lived with my parents and sister up until I was 18. But my sister was really abusive and physically violent. My mom is also unhoused; we have slept on the street together but not recently. |
|  | 18 – I experienced sexual assault from my father’s girlfriend’s son and also from a friend. Dad’s alcohol use got worse and I ended my relationship with him. I moved from [city] to [city] at 18, found a shelter where I stayed for six months. |
|  | 19 – I haven’t felt financially stable in a long time. I slept in my car in the Bay then got to a shelter in [city], had my own place for a bit through a transitional living program. Came back to [city] because my mom and sister were here but felt mentally unsafe with them, so I went to the shelters. |
|  | 25 –I lived with my mom until I was 18 and then I was kicked out. I was able to finish high school. I lived in a motel and car with my boyfriend for a while until the [shelter], where we stayed for 30 days. |
|  | 27 – I lived with my parents in L.A. then was sent to Utah for a program at 15. I lived on my own, then saved enough money to come back. I’m now in a transitional living program. |
| Immigrated | 1 – I immigrated from Russia after graduating high school. I encountered some difficulties and moved to [city] where I found housing through a church. I’m staying at [shelter] pursuing political asylum. |
| Unspecified reasons for housing instability | 6 – Currently living in room and board in [city]. |
|  | 8 – Moved around a lot as a child, had a hard time staying in one place. I’m currently in between shelters. |
|  | 10 – My parents split when I was 3, and I moved in with Dad. Mom took me at age 8-9, then Dad took me back when I was 16. I didn’t feel safe at home (violence) and experienced sexual abuse but Mom denies this. I lived in a homeless camp for about 6 months once. |
|  | 12 – Housing was stable until I was 18. Then I graduated high school. |
|  | 17 – I was put in foster care at 8 years old and had to move around a lot. I currently don’t have housing, I slept at a friend’s house recently. |
|  | 26 – Before I was at [shelter], my mom and I moved around in and out of shelters. |
