# Supplementary material for: Trauma and resilience in an urban clinic for unhoused young adults: A mixed methods study
Source: PLOS Ment Health. 2025 Sep 5;2(9):e0000392. doi: 10.1371/journal.pmen.0000392 (PMC12798583; doi:10.1371/journal.pmen.0000392)
Supplement: Attachment — Submitted filename: PLOS.Response Letter.docx [file pmen.0000392.s003.docx]

Trauma and Resiliency in an Urban Clinic for Unhoused Young Adults: A Qualitative Study

Response to Reviewers

Dear Reviewers,

Thank you for your thorough and detailed appraisal of our work. As a team, we have endeavoured to present our data with as much clarity as possible. We fortified our study for publication by making significant changes to all sections, most of which can be found on our “Tracked Changes” document. Please refer to the “Final” document for your review.

Of note, we have added a Dedication message for the unexpected passing of Machelle Wilson, one of our biostatisticians and original co-authors. We leave its placement in the article to your discretion.

In the following, we will directly respond to peer reviewer comments:

Peer Reviewer #1

| **Comment** | **Response** | **Reference Lines** |
| --- | --- | --- |
| 1. The authors write on page 6 (lines 118-119) that they used "quantitative statistics including chi-square, MEANS procedure, NPAR1WAY procedure, and the Kruskal-Wallis test." However, they do not report any statistics. I believe that statistics should be presented to improve strenght and quality of this manuscript. Authors can compare ACE participants in terms of depression, generalized anxiety, and resilience. Since all of these variables are continuous, Mann-Whitney U-test should be used for this purpose. The subjects should be divided into 2 groups - with higher and lower ACE experience, using a cut-off of 4 (based on the literature), or based on the mean or median. In addition, the association between categories of ACEs, depression, generalized anxiety, and psychological resilience should be compared with demographic variables (including gender, age, race, and housing situation) using the Pearson Chi-square test of independence. | **This recommendation has been implemented. The section describing our statistical methods has been significantly edited to include much greater detail about the analyses conducted. Mann-Whitney U, Pearson Chi-Square, and Fisher’s Exact tests have been described and conduced to identify differences between those with high vs. low ACES and those with high vs. low resilience.**  **The established cut-off of 4 for ACES classification was not used (the median was used instead) because of the high levels of ACES in this sample (a cut-off of 4 would not have allowed us to compare between high and low ACES groups, since the lowest score reported was a 4).** | 123-152 |
| 2. I suggest to add more data to Table 1, including ACE and resilience categories. | **This has been done.** | Table 1 |
| 3. Always report the mean, median, and standard deviation as basic subject characteristics in the results section. | **This has been done.** | Table 1 |
| 4. Methods and appendix 1 does not include GAD-7, while it is included in Table 1. Please add the missing information. | **The GAD-7 has been completed removed from this manuscript. There were very few valid responses to this questionnaire and it was not contributing meaningfully to our presentation of the results.** | n/a |
| 5. Each abbreviation in the figures should be explained in a note below the figure (e.g., ACES, TLF). | **This has been done.** | n/a |
| 6. It would be worthwhile to add an Excel file with the raw data, as recommended by the journal, to the supplementary materials. | **We decided to omit the raw data as most of it is reported in the paper as is and we are not sure how much it would meaningfully add to the publication as a whole. If important to the journal, we will contact our IRB for permission to share this data.** | n/a |

Peer Reviewer #2

| Introduction 1. The use of a direct quote from a study participant is engaging and humanises the topic. However, it is not immediately clear how this quote connects to the broader discussion on youth homelessness, adversity, and resiliency. Consider briefly introducing the relevance of the quote before presenting it to help the reader understand its significance. | **Removed quote as we agree it was not clearly connected to the opening ideas of our work.** | n/a |
| --- | --- | --- |
| 2. The transition between topics (youth homelessness, ACEs, resiliency, and the study’s purpose) could be smoother. For example, the paragraph on ACEs jumps from childhood trauma to epigenetic theories of disease transmission, which, while relevant, feels abrupt. A clearer bridge between ACEs and resiliency would improve coherence. | **We revised this paragraph as suggested by the reviewer to sequentially detail the effects of ACES during the childhood/adolescent period and the later effects in adulthood. We removed the comment on epigenetic theories since we felt it distracted from the main thread of this paragraph.** | 60-65 |
| 3. The definitions of ACEs and resiliency are well-integrated, but some sentences are overly detailed or redundant. Example: "ACES also impact the youth and adolescent years with links to developmental and educational delays, obesity, asthma, sleep issues, frequent infection, and higher levels of anxiety and depression." The list is extensive and somewhat repetitive given the earlier mention of poor physical and mental health outcomes. | **We revised this paragraph to be more succinct with less repetition.** | 60-65 |
| 4. The paragraph about the research team's involvement in a youth center is valuable but could more clearly justify why this study is necessary beyond personal experience. Consider specifying gaps in existing research or explaining why a qualitative approach is particularly useful in this context. | **As suggested by the reviewer, we expanded on our thoughts as to why a qualitative approach is useful for impacting policy, reform, and healthcare in this line of work.** | 74-79 |
| 5. The final paragraph presents the study’s aim well but could be more impactful. Instead of saying the study "generates hypotheses for care advancement," specify how findings could inform policy, intervention programs, or clinical practice. | **We modified the final paragraph to clarify that our study holds potential implications for policy reform and program design, serving as a model for other clinics and organizations seeking to better understand and meet the unique needs of this community. “Ultimately, we hope to influence both practice and policy in ways that prioritize long-term wellness, housing stability, and equitable access to comprehensive care.”** | 81-89 |
| Methods 1. While verbal consent is noted, the reason for using verbal rather than written consent is not explained. | **Since our patients were attending medical appointments at our clinic, we felt it was more practical and appropriate to obtain verbal consent. This method of consent was approved by our IRB.** | 92-95 |
| 2. The recruitment process only includes TAY checking into the clinic, which may exclude unhoused youth who do not seek clinic services. Acknowledge this limitation and discuss how it might affect generalisability. | **We included a sentence clarifying thiis in the Limitations section: “Most significantly, our data potentially excludes a substantial population of unhoused young adults who avoid formal services entirely. This population may differ systematically from our participants in important ways, including trauma exposure, coping strategies, trust in institutions, or severity of mental health challenges. Their exclusion limits our understanding of the full spectrum of experiences among unhoused youth.”** | 428-432 |
| 3. The study mentions the use of both qualitative and quantitative analyses; however, the methodological details for both are insufficient. Please provide a clearer description of the thematic analysis process, including coding strategies, how themes were identified, and steps taken to ensure rigour (e.g., interrater reliability). Additionally, elaborate on the quantitative analysis by specifying the statistical tests used, the rationale behind their selection, and how they were applied to the data. | **As mentioned previously, the section describing the statistical methods has been majorly edited to include much greater detail about the analyses conducted.** | 125-135 142-152 |
| 4. It is unclear how participants were categorised into low, medium, and high resiliency (e.g., cutoff scores). | **Thank you for this comment as a similar question was raised by Reviewer #1. Instead of using a three-level categorical variable, a two-level categorical variable was chosen for use in the current version of the manuscript. A description of how this was created (using a median cut-off) is now described in the Methods section.** | 146-152 |
| 5. The final sentence repeats information about IRB approval and verbal consent unnecessarily. | **This sentence was removed to minimize repetition.** | n/a |
| Results 1. The study mentions associations between housing status, PHQ-9, and resiliency scores, but does not report statistical significance. If statistical tests (e.g., chi-square, Kruskal-Wallis) were conducted, report p-values and effect sizes to support claims of relationships. If findings are exploratory, explicitly state that no statistically significant relationships were found. | **The methods section now describes the statistical tests implemented and the results consist of relevant descriptive statistics and the corresponding p-value to support these associational findings.** **Although no statistically significant relationships were found, in this small descriptive sample of TAY at our clinic, we found the insights clinically meaningful.** | 175-182 |
| 2. The study divides participants into low, medium, and high resiliency but does not explain: Why those score ranges were selected (e.g., based on prior research or distribution of scores?). Whether these categories were validated in previous studies. | **Instead of using a three-level categorical variable, a two-level categorical variable was chosen for use in the current version of the manuscript. A description of how this was created (using a median cut-off) is now described in the Methods section.** | 146-152 |
| 3. The qualitative themes are well-structured, but the coding process is not referenced in this section. Briefly mention how themes were identified (e.g., inductive vs. deductive coding, number of coders, intercoder agreement). | **As suggested by the reviewer, we expanded on our thematic coding process within the Methods section. We are happy to provide the raw data spreadsheet with our assigned codes if needed.** | 25-135 |
| Discussion  1. While the discussion is descriptive, it lacks comparative engagement with prior research. Incorporate references to other studies on homelessness, resilience, or trauma to contextualize findings. How do these findings compare with resilience levels in other homeless populations (e.g., adults, families)? Are similar trends observed in other geographic regions or demographic groups? | **We have now incorporated a variety of literature on youth homelessness and resiliency research that we believe enhances and engages with our discussion. For example, we speak about differences in familial connections between independent homeless youth and youth in homeless families.** | 345-350  352-357 |
| 2. The paragraph states, “Innate qualities such as resiliency are challenging to quantify.” This implies that resilience is entirely innate, which is debatable, as resilience is also shaped by environmental and social factors. Reframe this to acknowledge both inherent and learned components of resilience. ***** | **We introduce Zimmerman et al’s theory that resiliency is comprised of innate *assets* and external *resources* in the Introduction, and we added a sentence in our discussion to clarify our agreement that resiliency contains both elements.** | 360-362 |
| 3. The transition between topics (e.g., from trauma to resilience, and then to limitations) is somewhat abrupt. Introduce a clearer transition sentence between major sections. Consider structuring the discussion into subheadings (e.g., Key Findings, Interpretation, Implications, Limitations). | **When restructuring our discussion, we broke them into sub-headings to more precisely focus our findings and takeaways: Trauma Profiles, Resiliency Challenges, Housing Stability as a Resilience Catalyst, and Implications for Policy and Practice.** | 329, 359 etc |
| 4. The discussion touches on the role of TLPs in fostering resilience but does not elaborate on practical recommendations. Expand on policy implications by discussing: How can TLPs be improved to better support youth?  What interventions could help increase resilience among those with lower scores? | **We modified our discussion to expand on evidence-based improvement strategies for TLPs. TLPs and other resource programs for this population can focus on the strength of peer-based survival networks, including peer-based workshops and group education. Examples of such interventions are given in Table 4. We also engage with outside research arguing how social connections can drive positive change to an equal or even greater degree than basic need provision in homeless youth; therefore we emphasize that social connection, community, and professional therapeutic relationships deserve a crucial space for programming and funding in these programs.** | 417-424  Table 4 |
| 5. The text references Figure 3 but does not describe it in detail. Provide a brief explanation of what Figure 3 illustrates to ensure clarity for readers. | **We decided as a team to omit Figure 3 from this version of our work, since we decreased the number of resiliency categories from three to two.** | n/a |
